# Prenatal Diagnosis of Fetal Jejunal Atresia: A Case Report

**DOI:** 10.7759/cureus.18947

**Published:** 2021-10-21

**Authors:** Apostolia Galani, Athanasios Zikopoulos, Lampros Papandreou, Eirini Mastora, Konstantinos Zikopoulos, George Makrydimas

**Affiliations:** 1 Obstetrics and Gynaecology, University Hospital of Ioannina, Ioannina, GRC; 2 Obstetrics and Gynaecology, Royal Cornwall Hospital, Cornwall, GBR; 3 Fetal Medicine, University Hospital of Ioannina, Ioannina, GRC

**Keywords:** obstetrics and gynecology, ultrasound diagnosis, surgical management of atresia, intestinal atresia, jejunal atresia

## Abstract

Intestinal atresia is the result of fetal bowel maldevelopment which leads to congenital bowel obstruction. It is a common cause of ileus of the newborn and can occur at any site of the gastrointestinal tract. Prenatal diagnosis relies on the demonstration of dilated loops of the fetal bowel and the presence of polyhydramnios at the end of the second or more frequently the third trimester of pregnancy. This condition requires surgical correction soon after birth, with timely diagnosis improving the prognosis. Here, we present the case of a fetus diagnosed with jejunal atresia at 33weeks of pregnancy.

## Introduction

Neonatal intestinal atresia is a rare condition with a reported incidence of 1.3 to 3.5 per 10,000 live births. Although the pathophysiology is not completely understood, bowel atresia is most probably the result of a vascular accident in the corresponding branches of the mesenteric arteries. Neonates with bowel atresia need appropriate medical care soon after birth. If these neonates remain undiagnosed without treatment, their condition may deteriorate rapidly [1]. Thus, prenatal diagnosis is crucial. Neonates with bowel atresia should be delivered in hospitals with neonatal units capable of providing appropriate care, which significantly improves the outcome. However, ultrasound diagnosis is not feasible at the time of routine anomaly scan in the mid-trimester. The ultrasound signs are present beyond 24-26 weeks of gestation. In some cases, for example, in anal atresia, ultrasound diagnosis during pregnancy is difficult [2,3].

Surgical correction is performed as soon as the general condition of the neonate is stabilized and other malformations or chromosomal abnormalities are excluded. The postoperative mortality in cases without other anomalies is low. However, the outcome depends on the gestational age at birth, the exact position, and the size of the defect in the bowel. These neonates are also at risk for complications such as sepsis and short bowel syndrome [4].

Here, we present a case of jejunal atresia which was diagnosed at 33 weeks of gestation. Fetal chromosomal and structural anomalies were excluded before birth and the neonatologists were informed appropriately.

## Case presentation

A 35-year-old pregnant woman (G2P1), at 33 weeks of gestation, presented to the emergency department of our hospital reporting fluid leakage from the vagina. Her pregnancy so far was low risk. She was not on any regular medications, did not have any known allergies, and was a nonsmoker. Vaginal speculum examination revealed amniotic fluid leakage from the cervical os, and a diagnosis of preterm rupture of membranes was made.

**Figure 1:**
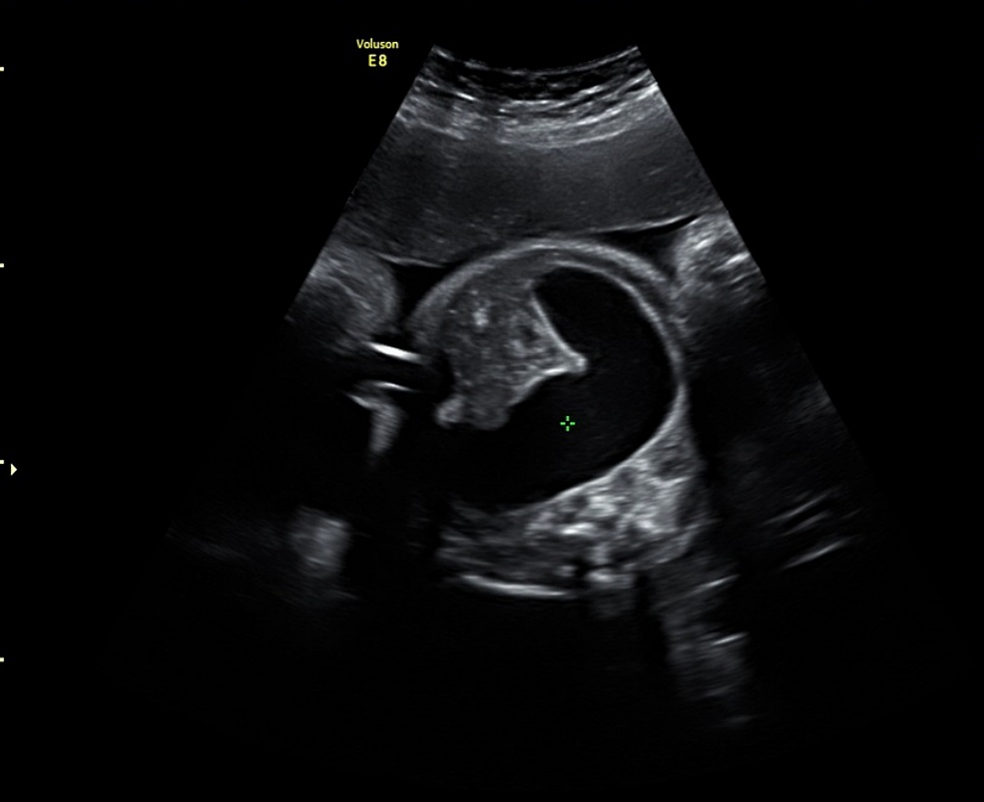
Distended bowel loops of the small intestine.

A transabdominal ultrasound examination revealed a normally grown fetus. Polyhydramnios was noted, with the deepest vertical pool measuring 10 cm. In addition, we noted small bowel dilatation, with a maximum diameter of 25 mm (Figure 1). These findings were compatible with fetal jejunal atresia. Detailed ultrasound examination demonstrated no other fetal malformations. A nuchal translucency scan at 12 weeks had shown a low risk for chromosomal abnormalities, and the anomaly scan at 22 weeks was normal.

The parents were counseled in detail about the diagnosis of fetal intestine atresia, the possible association with other abnormalities, and the postnatal management and prognosis. An amniocentesis was offered at 33+1 weeks for the exclusion of chromosomal abnormalities, and the parents were also tested for cystic fibrosis. The procedure was performed the following day at 33+2 weeks, and the results (karyotype, array-based comparative genomic hybridization [CGH]) were normal.

**Figure 2:**
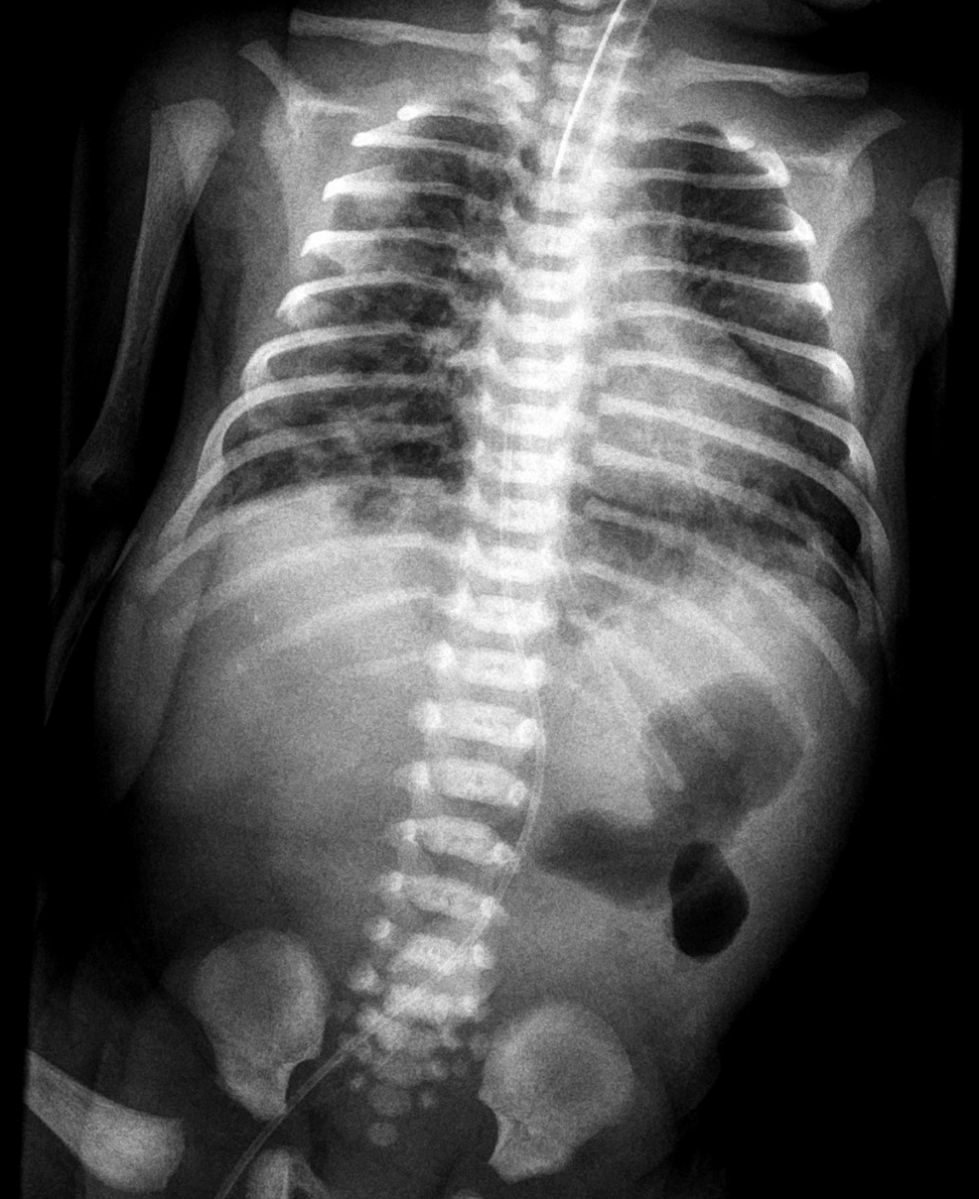
X-ray image post-delivery confirming the dilated loops.

At 34+5 weeks of gestation, she delivered a female neonate (2,680 g) by cesarean section because of initiation of labor with a history of a previous cesarean section. After birth, the neonate was examined by an expert neonatologist who found a mild abdominal distention with palpable distended loops of bowel. Appropriate medical treatment and parenteral nutrition were started the same day. The newborn underwent X-ray imaging according to the instructions of the neonatal team, and the following surgical management confirmed the diagnosis of jejunal atresia (Figure 2). Multiple atresias were found in approximately 70 cm of the jejunal. Following the confirmation of diagnosis, the entire section of the small bowel was resected and end-to-end anastomosis was performed. The condition of the infant after the operation was excellent and she was discharged after three weeks.

## Discussion

Bowel atresia may occur at any site of the small bowel or the colon. The most common type is duodenal atresia, which accounts for approximately 60% of the cases and is associated with cardiac renal and defects in 10-20% of the cases. In approximately 30% of the cases, a chromosomal abnormality is noted, mainly trisomy 21. Anorectal atresia is less common, and in up to 70% of the cases, urogenital, vertebral, and central nervous system abnormalities are noted. Chromosomal abnormalities are found in 4% of the cases, mainly trisomy 13 and 18 [2,3].

Jejunal or ileal atresia occurs in about 1 per 5,000 births, representing approximately 20% percent of small intestinal atresias [2-4]. Chromosomal abnormalities are less common but are associated with other bowel abnormalities, mainly malrotation and gastroschisis. There is a 10% risk of cystic fibrosis, and in these cases, meconium peritonitis may occur.

Several case reports and small studies have reported the feasibility of prenatal diagnosis of fetal bowel obstruction. However, the detection rate in these studies varies considerably between 10% and 90%, depending on the gestational age at the time of the ultrasound examination, the site of the obstruction, the presence of polyhydramnios and other structural abnormalities, the degree of the bowel dilatation, and the experience of the operator [4,5].

The diagnosis of anal atresia relies on the demonstration of dilatation of the rectum and the sigmoid in the third trimester while the amount of amniotic fluid remains normal. Most of the cases with no associated anomalies remain undiagnosed. On the contrary, duodenal atresia is commonly detected prenatally by the presence of the “double-bubble” sign and the presence of polyhydramnios beyond 24 weeks of gestation [6].

However, prenatal diagnosis of jejunal and ileal atresia is challenging. The fetal abdominal circumference may be enlarged, and the ultrasound diagnosis is based on the demonstration of multiple dilated loops of bowel >7 mm in diameter and mural thickness of >3 mm, with intense peristalsis, polyhydramnios, and, in cases of perforation of the bowel, ascites and meconium peritonitis. Increased echogenicity of the bowel may also be present, especially ιn fetuses affected by cystic fibrosis [7].

In fetuses with jejunal obstruction, there is a small number of dilated loops of the bowel, as seen in the current case (Figure 1), while multiple dilated bowel loops occur more often in ileal obstruction. Prenatal diagnosis of the size of the affected bowel or the presence of multiple lesions is difficult. Polyhydramnios is usually seen in cases of jejunal obstruction (about 50%) and rarely in cases with more distal obstruction.

Nevertheless, a recent systematic review reported an overall detection rate for jejunal and ileal atresia of 66.3% (95% confidence interval [CI] = 33.9-91.8%) and 25.9% (95% CI = 4.0-58.0%), respectively [6]. In addition, they reported that both dilated loops of bowel and polyhydramnios provided a low overall detection rate. However, in a subanalysis considering only the most recent studies (published in the last 10 years), they did not find any improvement in the detection rate of bowel atresia. The authors of this systematic review concluded that the diagnostic performance of ultrasound in identifying bowel atresia is extremely variable and further large-scale studies with specific criteria are needed.

In the current case, the diagnosis was made because of polyhydramnios and the presence of a small number of dilated loops of the bowel. Both findings are typical of proximal bowel obstruction. A detailed ultrasound scan was performed to exclude other malformations, mainly abnormalities of the gastrointestinal system. The parents were tested for cystic fibrosis, and, although this condition is not associated with increased risk for chromosomal abnormalities, amniocentesis was performed and array-based CGH was normal.

In this case similar to other prenatally diagnosed cases, the diagnosis was confirmed with plain radiographs of the abdomen soon after birth, showing dilated loops of the small bowel with air-fluid levels. Contrast studies can also be performed to localize the defect prior to the surgical treatment.

In neonates with bowel obstruction, a nasogastric tube is placed for suction of the gastric secretions, and parenteral nutrition must be initiated. Immediate surgery is recommended in cases complicated by malrotation and volvulus. In other cases, the procedure can be postponed until metabolic or other coexisting abnormalities are treated appropriately [8].

A common problem in babies with jejunal atresia is that the segment of the bowel proximal to the obstruction becomes dilated because of the intense peristalsis and may cause problems with anastomosis. Additionally, it may lead to functional obstruction after the repair due to ineffective peristalsis. To avoid this complication and minimize dysfunction at the site of the anastomosis, some surgeons taper or resect the most dilated proximal bowel [5,6].

Primary repair is preferred if multiple atresias are present, and care should be taken to preserve intestinal length, ideally more than 75 cm, to avoid short bowel syndrome which is a lethal condition [6-8].

## Conclusions

Prenatal diagnosis of small bowel atresia has an overall sensitivity of approximately 50%, whereas two-thirds of the cases of jejunal atresia can be diagnosed. This case illustrates the potential of the diagnosis of proximal obstruction because of the presence of marked bowel dilatation and polyhydramnios. Prenatal diagnosis is crucial because parents can be counseled appropriately, detailed examinations can be performed to exclude other malformations or syndromes, and delivery can be arranged in a center with appropriate facilities.
